# A single-cell trajectory atlas of striatal development

**DOI:** 10.1038/s41598-023-36255-5

**Published:** 2023-06-03

**Authors:** Ashley G. Anderson, Ashwinikumar Kulkarni, Genevieve Konopka

**Affiliations:** 1 Department of Molecular and Human Genetics, Baylor College of Medicine, Houston, TX 77030 USA; 2 Department of Neuroscience, UT Southwestern Medical Center, Dallas, TX 75390-9111 USA; 3 Peter O’Donnell Jr. Brain Institute, UT Southwestern Medical Center, Dallas, TX 75390 USA

**Keywords:** Cell fate and cell lineage, Genetics of the nervous system, Developmental disorders

## Abstract

The striatum integrates dense neuromodulatory inputs from many brain regions to coordinate complex behaviors. This integration relies on the coordinated responses from distinct striatal cell types. While previous studies have characterized the cellular and molecular composition of the striatum using single-cell RNA-sequencing at distinct developmental timepoints, the molecular changes spanning embryonic through postnatal development at the single-cell level have not been examined. Here, we combine published mouse striatal single-cell datasets from both embryonic and postnatal timepoints to analyze the developmental trajectory patterns and transcription factor regulatory networks within striatal cell types. Using this integrated dataset, we found that dopamine receptor-1 expressing spiny projection neurons have an extended period of transcriptional dynamics and greater transcriptional complexity over postnatal development compared to dopamine receptor-2 expressing neurons. Moreover, we found the transcription factor, FOXP1, exerts indirect changes to oligodendrocytes. These data can be accessed and further analyzed through an interactive website (https://mouse-striatal-dev.cells.ucsc.edu).

## Introduction

The striatum is a highly conserved forebrain structure important for regulating a wide range of motor and cognitive behaviors^1^. This region receives dense glutamatergic and neuromodulatory inputs from several brain regions, including the cortex, thalamus, and substantia nigra^1^. These diverse inputs are integrated and propagated to downstream basal ganglia nuclei via distinct classes of striatal GABAergic spiny projection neurons (SPNs), interneurons, and glial cell types. Disruption of striatal cell types has been observed across several neurodevelopmental and neurodegenerative disorders, including autism spectrum disorders (ASD) and Huntington’s disease (HD)^2,3^. Uncovering the molecular mechanisms regulating striatal cell type development in the brain is therefore an important step towards identifying mechanisms altered in disease states to ultimately improve therapeutics.

High throughput single-cell RNA-sequencing (scRNA-seq) technology has advanced our understanding of the cellular composition and molecular characterization of the brain^4^. In the striatum specifically, recent scRNA-seq studies have led to important insights into striatal cellular composition and further unraveled the molecular differences between the principal striatal spiny projection neurons and aspiny interneuron subtypes^5–11^. SPNs and interneurons are derived from separate progenitor pools from either the lateral ganglionic eminence (LGE) or medial and caudal ganglionic eminence (MGE, CGE), respectively. SPNs are classically divided into neurons that express dopamine receptor-1 receptor (D1) and project along the *direct* pathway (dSPNs) or SPNs that express dopamine receptor-2 (D2) and project along the *indirect* pathway (iSPNs)^1^. scRNA-seq studies have found more diversity among SPN subtypes than previously appreciated, including a small population of neurons that express either D1 or D2 receptors but have distinct molecular profiles from canonical SPNs (“eccentric” SPNs, or eSPNs)^9^. This population was masked by previous studies relying on fluorescent-reporter-driven techniques^12^ to identify and separate dSPNs versus iSPNs for molecular characterization followed by bulk RNA-sequencing approaches, showing the importance of single-cell methodologies^13–15^. Striatal scRNA-seq studies have also shed light on the cell type specific molecular changes that occur upon disrupting genes important for striatal development, such as *FOXP1*, a gene strongly associated with autism and intellectual disability in humans^5^. Though highly expressed in both dSPNs and iSPNs, deletion of *Foxp1* severely affected iSPNs and significantly reduced that cellular population specifically^5^. Striatal interneurons make up ~ 5% of the striatal neuron population and are largely divided into subtypes that include *Pvalb*-expressing, *Sst/Npy/Nnos*-expressing, *Calb2*-expressing, and *Th*-expressing groups^8,16^. Single-cell studies of striatal interneurons have found interneurons subgroups that are molecularly discrete (i.e. *Npy*^+^*/Sst*^+^, *Chat*^+^, *Th*^+^, *Npy*^+^*/Sst*^*−*^, *Cck*^+^) or display continuous gradients of gene expression (*Pvalb*)^8^. While these studies have furthered our understanding of striatal cellular and molecular development, each study was performed at a single time point and the developmental trajectory of striatal cell types spanning development remains incomplete. A recent single-cell study has made some progress along these lines by profiling cells from the human fetal lateral ganglionic eminence from 7 to 11 post conceptual weeks and identified key transcription factors important for governing D1 and D2 lineage specification, highlighting the benefit of trajectory-level analyses^17^.

To identify the key molecular mechanisms spanning striatal development, we combine previously published single-cell or single-nuclei RNA-sequencing datasets at distinct embryonic and postnatal timepoints in the mouse brain to build a striatal cell type-specific developmental trajectory map^5–11^. From this integrated dataset, we investigate the trajectory pattern and gene regulatory networks within both neuronal and glial populations. We find that dSPNs and iSPNs diverge in their postnatal pseudotime trajectory, with dSPNs exhibiting greater transcriptional complexity compared to iSPNs. We further show how interneuron subtypes and oligodendrocytes change their molecular composition over development. Moreover, we show that FOXP1 may indirectly alter oligodendrocyte maturation via SPN-specific disruption. We created an interactive website to easily access and further analyze these datasets. This resource is an important step towards compiling single-cell data from across labs and methodologies to further our understanding of neural development.

## Results

### Combined striatal single-cell datasets across development

**Figure 1:**
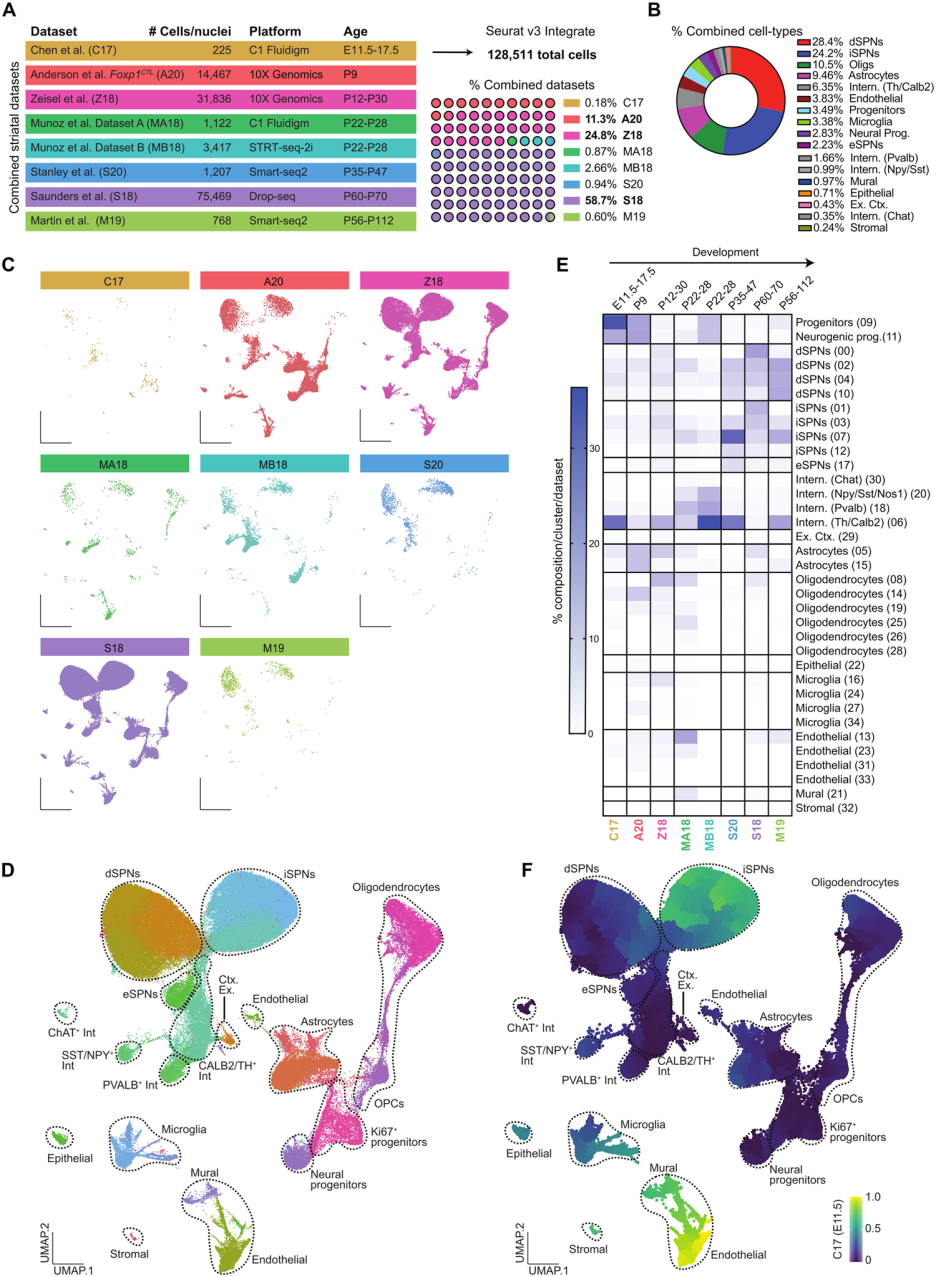
Integrated striatal single-cell datasets across timepoints and single-cell methodologies. (**A**) Table describing the published datasets integrated in our analysis and the percent composition of each study to the combined dataset. (**B**) The percent composition of cell types within the combined dataset. (**C**) UMAP plots showing where cells from each dataset clustered in the combined analysis. (**D**) UMAP of combined dataset colored by cluster affiliation and annotated with cell type identification. (**E**) The percent contribution of cells per cluster (35 total) for each dataset ordered by developmental time. (**F**) Pseudotime analysis using Monocle3 of the combined dataset plotted with UMAP coordinates.

To build a single-cell developmental trajectory map of striatal cell types, we integrated previously published striatal single-cell datasets from seven studies that collected data from mouse brain at different timepoints during striatal development (Fig. 1A). These data include single cells from medial and lateral ganglionic eminences and mature striatal tissue between the ages of embryonic day (E) E11.5-E17.5^6^ (C17, 225 cells), postnatal day (P) P9^5^ (A20, 14,467 cells), P12-P30^11^ (Z18, 31,836 cells), P22-P28^8^ (Dataset A, MA18: 1122 cells and Dataset B, MB18: 3417 cells), P35-47^10^ (S20, 1207 cells), P60-P70^9^ (S18, 75,469 cells), and P56-112^7^ (M19, 768 cells). The number of genes detected per dataset was related to the number of cells sequenced, with more genes present in datasets with fewer cells that were more deeply sequenced using Smart-seq2 (C17, S20, and M19, Fig. S1A).

The combined dataset resulted in 128,511 total cells with 35 unique clusters (Fig. S1C). Three datasets across postnatal development contributed greater than 95% of cells to the combined analysis (Fig. 1A). dSPNs (28.4%) and iSPNs (24.2%) comprised ~ 52.6% of the total dataset, followed by oligodendrocytes (10.5%), astrocytes (9.5%), and interneurons (6.35%) (Fig. 1B). No clusters were unique to a given dataset (Fig. 1C and Fig. S1C), with cells clustering primarily by cell type identity (Fig. 1D and Fig. S1D). The number of genes were greater in neuronal cell types (Fig. S1B) or across neuronal clusters (Fig. S1C) as seen in previous single-cell studies of brain tissue^18^. We found that the percentage of cells from the embryonic and early postnatal timepoints were more abundant in the progenitor and neural progenitor clusters compared to P12-P112 timepoints (Fig. 1E). Four datasets used cellular isolation methods to enrich for distinct cell types, interneurons for MA18 and MB18^8^ and SPNs for M19 and S20^7,10^, which is observed in the percent composition of cell types within these studies (Fig. 1E).

To examine the differentiation trajectory pattern of the combined dataset, we used Monocle3^19^ to organize cells along a pseudotime scale by setting the root as the biologically earliest cells (E11.5). We then projected their pseudotime values onto UMAP coordinates (Fig. 1F). Using this method, we observed the most dynamic changes in cellular trajectory patterns within three cell types: SPNs, microglia, and vascular cells (Fig. 1F). Within the SPN population, a distinct change in trajectory pattern was observed between dSPNs and iSPNs, with iSPNs progressing faster along the differentiation trajectory compared to dSPNs. These findings suggest dSPNs and iSPNs have distinct developmental trajectory patterns.

### Extended period of gene expression dynamics in dSPNs relative to iSPNs

**Figure 2:**
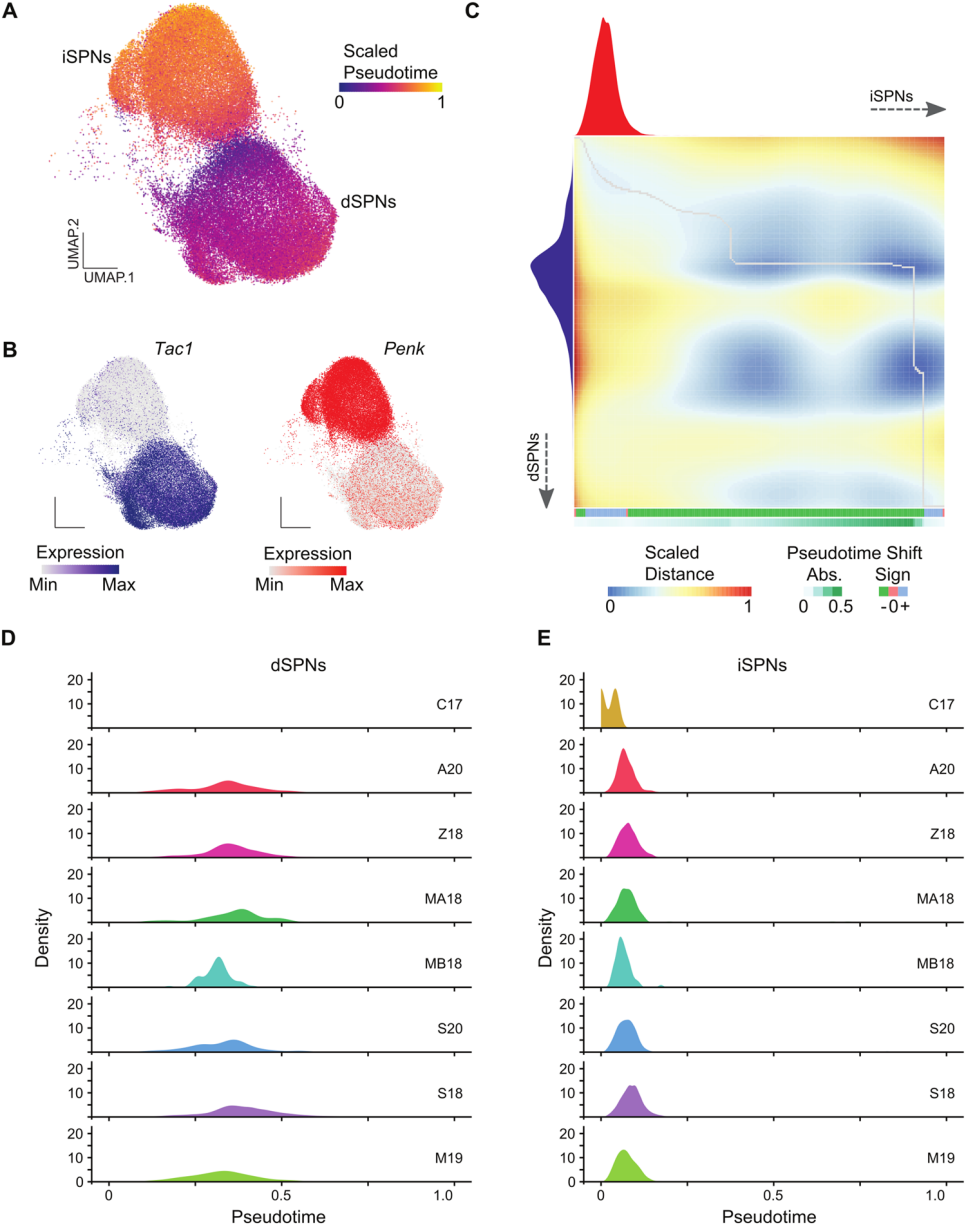
Comparing pseudotime trajectory dynamics between dSPNs and iSPNs. (**A**) UMAP plot colored by PHATE pseudotime scale with (**B**) feature plots showing the expression of dSPN marker (*Tac1*) and iSPN marker (*Penk*). (**C**) Dissimilarity matrix and global alignment of pseudotime trajectories between dSPNs (x-axis) and iSPNs (y-axis) with pseudotime shifts labelled below. (**D**) Plots of *Tac1* (dSPNs) or (**E**) *Penk* (iSPN) expression across pseudotime separated by dataset.

To further study the pseudotime trajectories of dSPNs versus iSPNs, we isolated SPNs from the combined dataset, using clusters 0, 2, 4, 10 for dSPNs and 1, 3, 7, 12 for iSPNs (Fig. 1E). We used PHATE^20^ to perform a pseudotime analysis only on SPNs (Fig. 2A). Similar to the results found using Monocle3^19^ on all cells, iSPNs were farther along in pseudotime compared to dSPNs (Fig. 2A,B). To quantitatively compare the trajectory dynamics between dSPNs and iSPNs we used cellAlign^21^ to compare single-cell pseudotime trajectories. The outputs of this analysis are a global alignment-based dissimilarity matrix and a pseudotime shift score indicating differences between pseudotime values (Fig. 2C). Using this method, we observed a distinct pseudotime shift between iSPNs and dSPNs, indicating that faster gene expression dynamics occur within iSPNs relative to dSPNs (Fig. 2C). This pseudotime shift hints at an extended period of gene expression dynamics occurring in dSPNs compared to iSPNs over postnatal timepoints (Fig. 2C). This change in pseudotime dynamics is observed across each dataset when plotting the expression of dSPN markers (*Drd1*, *Tac1*, Fig. 2D, Fig. S2A top panel) or iSPN markers (*Penk*, *Drd2*, Fig. 2E, Fig. S2A bottom panel) across pseudotime. We note that the embryonic C17 dataset has low signal for dSPNs. Therefore, we used the early postnatal (P9) cells to set the pseudotime trajectory root and observed the same pseudotime shifts, suggesting that this difference in relative gene expression dynamics occurs during postnatal development (Fig. S2B,C).

### dSPNs have more discrete transcriptional networks

**Figure 3:**
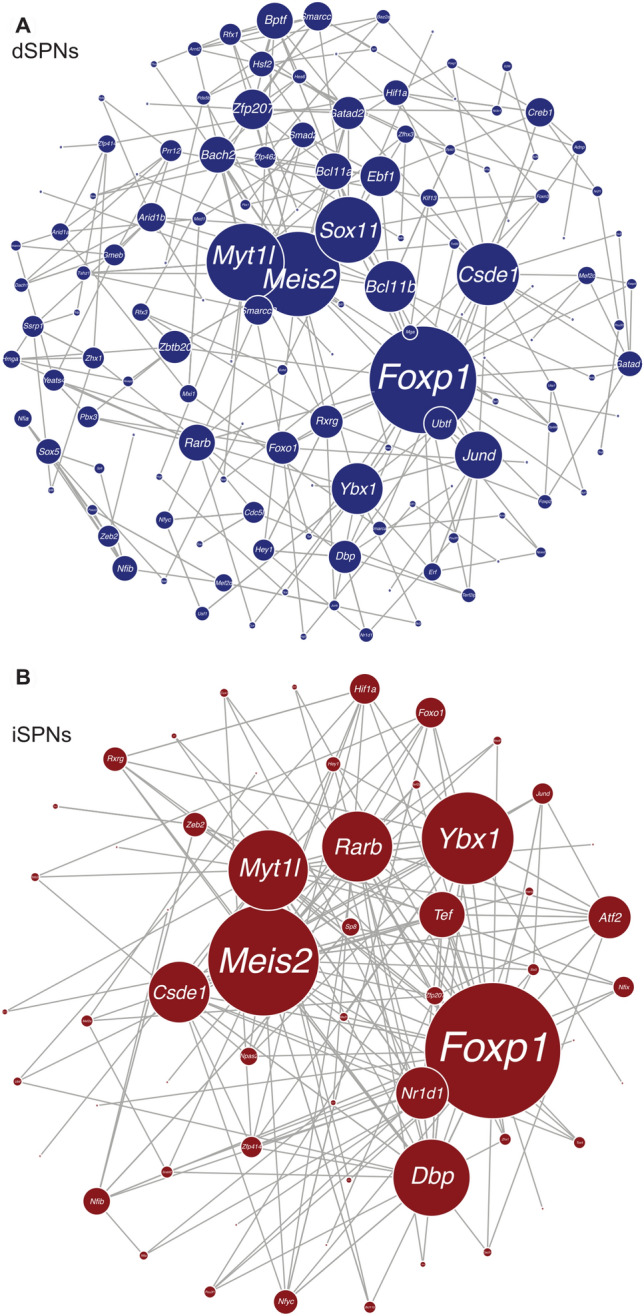
Gene regulatory analysis of dSPN and iSPNs. Visualization of network transcription factors in dSPNs (**A**) or iSPNs (**B**). Gray lines between hubs indicate degree of interconnectivity.

We next used a gene regulatory network (GRN) analysis to identify key transcription factors (TFs) involved in dSPN and iSPN development (Fig. 3). dSPNs and iSPNs have several shared hub TFs including *Foxp1*, *Myt1l*, *Meis2*, and *Csde1*. We also identified hub TFs unique to each subpopulation. dSPNs unique hub TFs included *Sox11*, *Bcl11b*, *Ybx1*, and *Ebf1* (Fig. 3A). iSPNs unique hub TFs included *Rarb*, *Nr1d1*, and *Tef* (Fig. 3B). We observed that dSPNs had more discreet transcriptional networks, compared to iSPNs whose hub genes were more interconnected. Moreover, the dSPNs TF hub genes were enriched with markers of early-born neurons, including *Sox4* and *Sox11* (Fig. 3A). These results suggest that dSPNs have more transcriptional complexity compared to mature iSPNs.

### Interneuron pseudotime trajectories over striatal development

**Figure 4:**
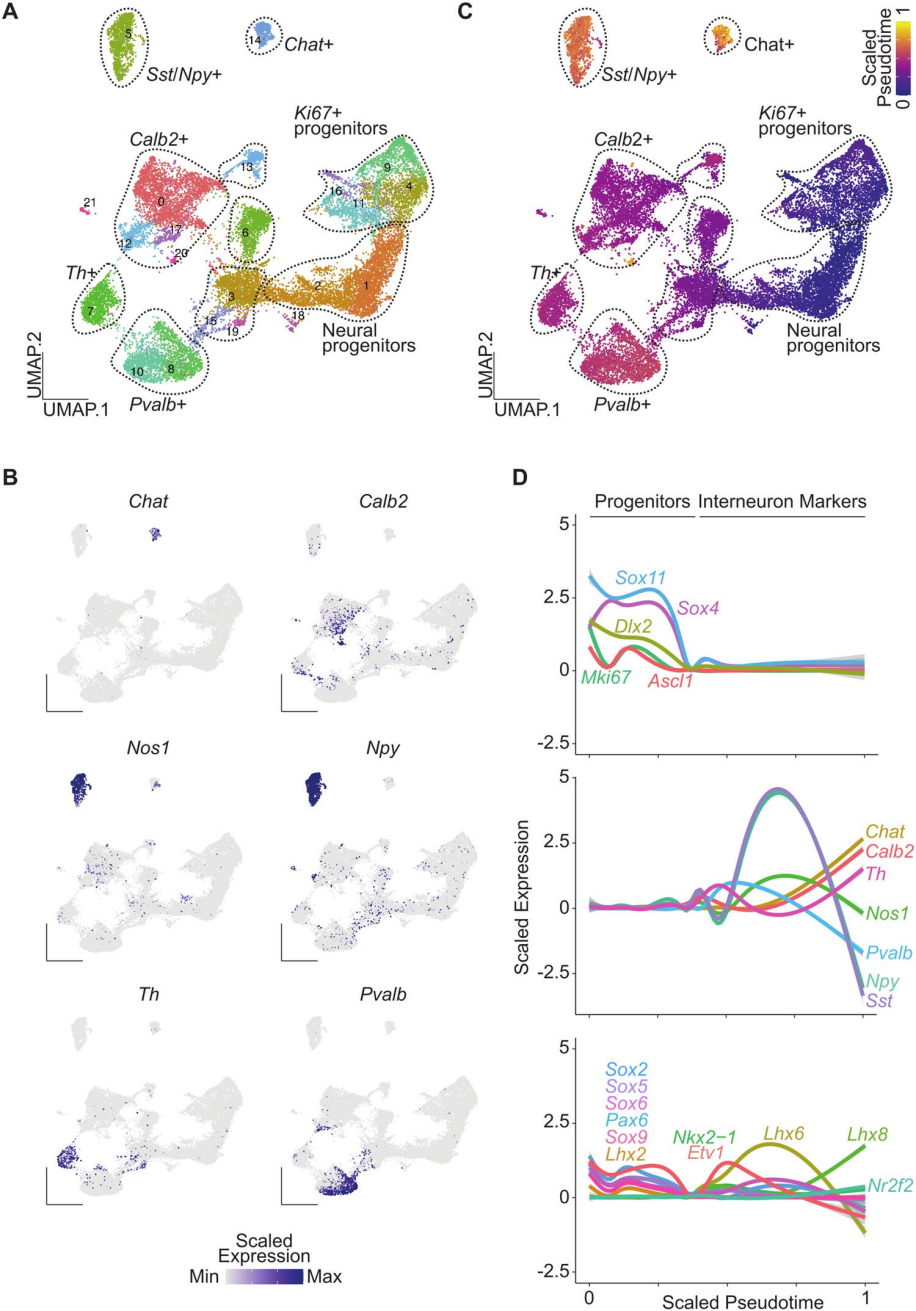
Pseudotime analysis of striatal interneuron subtypes. (**A**) Annotated UMAP clusters of interneuron and neural progenitor clusters isolated from the integrated dataset. (**B**) Scaled expression of genes enriched in distinct interneuron populations. (**C**) UMAP of cells colored by PHATE pseudotime values. (**D**) Expression of progenitor markers (top panel), interneuron subtype markers (middle panel), and key transcription factor important for interneuron development (bottom panel) across pseudotime.

Several interneuron subtypes populate the striatum and are critical for striatal function. We isolated both interneuron and neural progenitor clusters from the integrated dataset to analyze the pseudotime trajectory pattern of these interneuron subtypes (Fig. 4A). We identified interneurons by key molecular markers including *Chat* (cholinergic interneurons), *Npy* (Neuropeptide Y), *Nos1* (Nitric oxide synthase 1), *Sst* (somatostatin), *Pvalb* (Parvalbumin), *Th* (Tyrosine hydroxylase), and *Calb1* (Calbindin 1) (Fig. 4B). Several of these markers colocalize in the same cells (*Nos1*, *Npy*, *Sst*), whereas *Th*, *Pvalb*, *Calb1*, and *Chat* interneuron clusters were distinct. Using PHATE^20^, we found distinct differences in the pseudotime differentiation trajectory of interneuron subtypes (Fig. 4C). *Chat* and *Nyp*/*Sst* interneurons were further along in pseudotime compared to the other subtypes, followed by *Pvalb*, *Th*, and *Calb2* expressing interneurons (Fig. 4C). We next plotted the expression of key markers of the progenitor state (*Sox4*, *Sox11*, *Dlx2*, *Mki67*, *Ascl1*), interneuron markers, and the TFs highly associated with interneuron development (*Sox2/5/6/9*, *Pax6*, *Lhx2*, *Nkx2.1*, *Etv1*, *Lhx6/8*, and *Nr2f2*) (Fig. 4D)^22^. As expected, peak expression of progenitor markers occurred early in pseudotime, whereas interneuron markers peaked later in pseudotime with little to no overlap. We also saw that *Lhx6* and *Lhx8* expression peaked along the scaled pseudotime after *Nkx2.1* expression, since both are downstream of *Nkx2.1*. Moreover, *Lhx6* is critical for *Pvalb* and *Sst/Npy/Nos1* interneuron specification. *Lhx8* increased over pseudotime following the same trend as *Chat*, an expected finding given that *Lhx8* is important for *Chat* interneuron development and function. Interestingly, we found a bimodal pseudotime pattern of many TFs associated with interneuron development, suggesting successive or distinct waves of interneuron development. This patterning could potentially represent regional differences from interneurons derived from different subregions within the medial or caudal GE, since cell types from both regions have unique cellular trajectories^23^. These results indicate that interneuron subtypes develop along distinct trajectory patterns and provide a rich resource for researchers to further investigate molecular development of striatal interneurons.

### Oligodendrocyte pseudotime trajectories over striatal development

**Figure 5:**
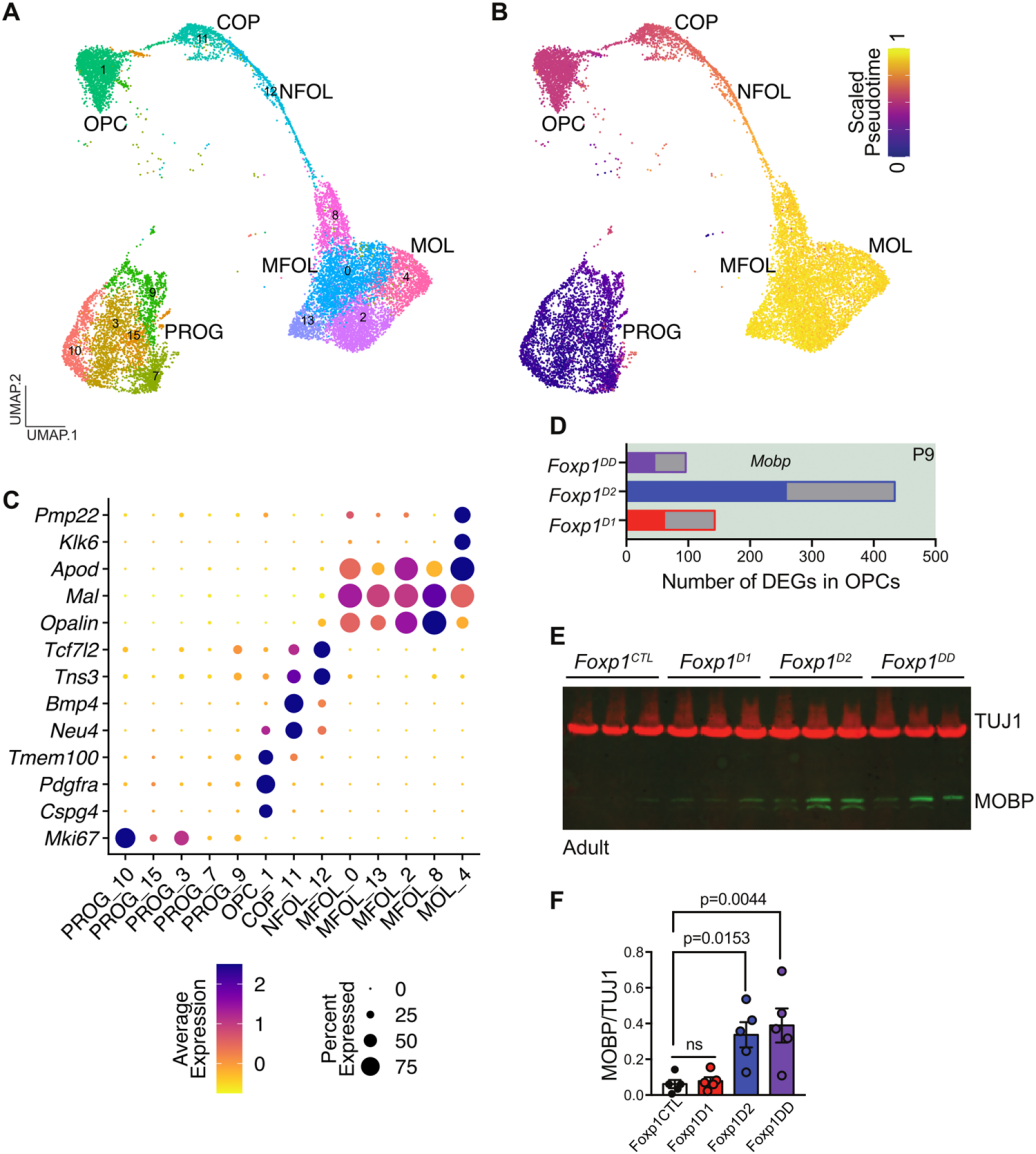
Pseudotime analysis of oligodendrocytes across striatal development and with deletion of *Foxp1*. (**A**) Annotated UMAP of oligodendrocyte progenitor cells (OPCs), committed oligodendrocyte precursors (COPs), newly-formed oligodendrocytes (NFOL), myelin-forming oligodendrocytes (MFOL), mature oligodendrocytes (MOL) and progenitor clusters from the combined dataset. (**B**) Cells colored by PHATE pseudotime values. (**C**) Scaled expression of genes specific to each oligodendrocyte subtype. (**D**) Number of differentially expressed genes (DEGs) in OPCs from P9 dataset with deletion of *Foxp1* from dSPNs, iSPNs, or both. Filled bars are upregulated DEGs (such as *Mobp*) and grey bars are downregulated DEGs. (**E**) Western blot of TUJ1 (housekeeping gene) and MOBP (oligodendrocyte marker) in striatal tissue in *Foxp1*^*CTL*^, *Foxp1*^*D1*^, *Foxp1*^*D2*^, and *Foxp1*^*DD*^ mice and (**F**) quantification of MOBP levels relative to TUJ1 across genotypes (N = 5/genotype). Data is represented as mean ± SEM. P-values determined using a one-way ANOVA with Dunnett’s multiple comparison.

We next wanted to examine the developmental trajectory of the second most abundant cell type within this integrated dataset, oligodendrocytes. We isolated both the oligodendrocyte and progenitor clusters from the integrated dataset and identified clusters for oligodendrocyte precursors (OPC, cluster 1), committed oligodendrocyte precursors (COP, cluster 11), newly-formed oligodendrocytes (NFOL, cluster 12), myelin-forming oligodendrocytes (MFOL, clusters 0, 2, 8 and 13), mature oligodendrocytes (MOL, cluster 4) along with progenitors (PROG, clusters 3, 7, 9, 10 and 15) (Fig. 5A). Using PHATE to obtain pseudotime trajectory values for each cell, we found distinct trajectory originating from progenitors (*Mki67*^+^) to clusters enriched for markers of mature oligodendrocytes (*Klk6*^+^, *Apod*^+^) progressing through OPCs, COPs, NFOL and MFOL (Fig. 5B). OPCs were marked by the expression of gene markers such as *Pdgfra* and *Cspg4* (Fig. 5C). Genes previously associated with astrocytes or radial glia (*Tmem100*) also appeared to be enriched in OPCs consistent with the origin of OPCs from radial glia-like cells and their ability to generate astrocytes in an event of injury^24,25^ (Fig. 5C). COPs were distinct from OPCs and expressed *Neu4* and genes involved in keeping oligodendrocytes undifferentiated such as *Bmp4*^25,26^ (Fig. 5C). NFOLs expressed genes involved in oligodendrocyte differentiation such as *Tcf7l2*^25,27^ (Fig. 5C). Both COPs and NFOLs also showed expression of genes involved in migration such as *Tns3*^25^ (Fig. 5C). MFOLs expressed genes such as *Mal* and *Opalin* known to be responsible for myelin formation whereas MOLs expressed late oligodendrocyte differentiation genes (*Klk6*, *Apod*) including genes enriched in myelinating cells (*Pmp22*)^25,28^ (Fig. 5C). These findings show that striatal oligodendrocytes have distinct subtypes with unique gene expression and trajectory profiles.

### Deletion of *Foxp1* upregulates oligodendrocyte marker MOBP in striatum

To better understand the non-cell autonomous effects of disrupting a key transcription factor in striatal SPN development (Fig. 3), we performed a pseudobulk differential gene expression analysis within oligodendrocytes in the P9 striatal single-cell dataset with *Foxp1* deleted from either dSPNs (*Foxp1*^*D1*^), iSPNs (*Foxp1*^*D2*^), or both (*Foxp1*^*DD*^). We found both upregulated and downregulated DEGs in oligodendrocytes across all genotypes, but more DEGs were observed when *Foxp1* was deleted specifically from iSPNs (Fig. 5D). The mature oligodendrocyte marker, *Mobp*, was upregulated in *Foxp1*^*D2*^ samples and we confirmed this finding at the protein level (Fig. 5E,F, Fig. S3). While deletion of *Foxp1* in iSPNs was shown to have non-cell-autonomous effects on dSPNs^5^, we now show that loss of *Foxp1* in iSPNs also exerts non-cell-autonomous effects on oligodendrocytes in the striatum.

## Discussion

The striatum is a hub for propagating signals from multiple brain regions to modulate complex learning and motor behaviors. Here, we have developed a single-cell transcriptome resource with the goal of increasing understanding of striatal molecular development at cellular resolution. We have developed an interactive website that integrates previously published striatal single-cell datasets across timepoints and technological modalities. This resource can also be expanded to include additional datasets and can be easily navigated by bench scientists.

Using this integrated striatal single-cell dataset, we analyzed trajectory information for the main neuronal cell types (dSPNs, iSPNs, and interneurons) and one major glial cell type (oligodendrocytes) of the striatum. Our findings suggest that dSPNs have greater transcriptional complexity compared to iSPNs during postnatal development. In line with our findings, a study of human embryonic striatal scRNA-seq found that dSPNs had slower differentiation kinetics compared to iSPNs and dSPNs had a greater number of transcriptionally distinct clusters^17^. This is interesting given the enrichment of dSPNs in distinct neurochemical compartments of the striatum, known as the striosome (or “patch”), compared to iSPNs. Striosomes receive dense dopaminergic innervation from the VTA and substantia nigra. This innervation becomes more dense over postnatal development, which might play a role in the different trajectory patterns and transcriptional states observed between dSPNs and iSPNs in our analysis.

The striatum contains a substantial population of oligodendrocytes and these cells likely constitute the increased amount of myelination that occurs postnatally on axon tracts targeting and passing through the striatum. Oligodendrocytes are responsible for generating myelin sheaths for the optimization of signal conductance, maturation, survival, and regenerative properties of axons. They are also vulnerable to dysfunction in numerous disorders, including ASD and HD. For example, oligodendrocyte density is increased within HD post-mortem striatum compared to healthy controls. A mouse model of Timothy syndrome, a severe congenital syndrome associated with autism and caused by mutations in an L-type voltage-gated Ca+ channel (Cav1.2), exhibits accelerated oligodendrocyte development and myelination in the striatum^29^. How oligodendrocytes mature in the striatum over development at the single-cell level is unknown. We found that striatal oligodendrocytes have a distinct lineage with different developmental stages.

Non-neurons, including oligodendrocytes, can send and receive signals to neurons. Such interactions are ultimately important for normal development and function of neurons. Single cell genomics can be harnessed to uncover non-cell autonomous effects on gene expression with the alteration of individual genes in specific cell types. Thus, we asked whether manipulation of striatal SPNs might alter non-neuronal populations in the striatum. We examined how deletion of the transcription factor *Foxp1*, a hub transcription factor in our GRN analysis of SPNs, alters the trajectory pattern of striatal oligodendrocytes. We identified non-cell autonomous gene expression changes in oligodendrocytes with deletion of *Foxp1* in dSPNs, iSPNs, or both cell types. Similar to the Timothy syndrome mouse model, we found that loss of FOXP1 specifically in iSPNs enhanced the maturation of oligodendrocytes and significantly increased the mature oligodendrocyte marker MOBP in adult striatum. These findings are just one example of how this resource can be queried to understand the role of individual genes on cell type specific patterns of expression over striatal development in both a cell autonomous and non-cell autonomous manner. Ultimately, this resource should further our understanding of striatal neurobiology at the single-cell level and aid in addressing therapeutic challenges facing neurodevelopmental and degenerative disorders that alter striatal function.

## Materials and methods

### Integration analysis

First, raw counts, matching cell type and meta information for each of the datasets was downloaded from respective sources. After checking the integrity of the datasets, raw counts for only common protein-coding genes across all the datasets were retained. Data processing and analysis was performed using R. Individual datasets were first filtered following cutoffs mentioned in each published paper (see table below). For dataset(s) with ‘NA’ cutoffs, either the datasets were already filtered and/or mitochondrial genes were already filtered out. Also, genes with no expression in any of the cells and genes from chromosomes X, Y and M were removed. Following the filtering, each dataset was processed through the standard Seurat (v3) pipeline (*NormalizeData*, *FindVariableFeatures*, *ScaleData*, *FindNeighbors*, *RunUMAP*, *FindClusters*) regressing for the number of UMIs and percent mitochondrial content (https://satijalab.org/seurat/archive/v3.0/pbmc3k_tutorial.html)^30^. Seurat objects for each of the datasets were then combined using Seurat’s integration (https://satijalab.org/seurat/archive/v3.0/integration.html)^30^ approach (*FindIntegrationAnchors*, *IntegrateData*) with 30 principal components. Data were clustered (*FindNeighbors*, *FindClusters*) using the original Louvain algorithm with a resolution of 0.8 and the clusters were visualized with Uniform Manifold Approximation and Projection (UMAP)^4,31^ in two dimensions (*RunUMAP*) for a total of 128,511 single cells or nuclei from the mouse striatum. Gene markers enriched for each cluster were identified using ‘*FindAllMarkers*’. Clusters were then annotated using the ‘*LabelTransfer*’ approach from Seurat (https://satijalab.org/seurat/archive/v3.0/integration.html)^30^ using cell types defined in S18 as reference.

### Pseudotime trajectory analysis for all cell types

The integrated Seurat object with all cell types for all datasets was converted into a Monocle (v3) compatible object using the ‘*as.cell_data_set*’ command. The Monocle object was then pre-processed (*cluster_cells*, *learn_graph*) using the standard Monocle pipeline^19,32^ (https://cole-trapnell-lab.github.io/monocle3/docs/trajectories/). Further, E11.5 cells from C17 were selected as the root population for performing pseudotime trajectory analysis (*order_cells*). UMAP plots colored by scaled pseudotime values were then generated.

**Table:** 

Dataset	# UMI cutoff	% Mito cutoff	# Cells
C17	3,000,000	NA	225
A20	≤ 50,000	≤ 10	14,467
Z18	NA	≤ 10	31,836
MA18	≤ 20,000	NA	1122
MB18	≤ 20,000	NA	3417
S20	NA	NA	1207
S18	≤ 40,000	≤ 10	75,469
M19	NA	NA	768
Total			128,511

### SPN sub-clustering and pseudotime trajectory analysis

Cell barcodes corresponding to clusters annotated as SPNs (dSPNs, iSPNs and eSPNs) were then used to subset the SPN population from all the cells. Using raw counts corresponding to the identified SPN population for all datasets were then used to run the Seurat integration approach (*FindIntegrationAnchors*, *IntegrateData*) to identify sub-populations among major SPN categories (https://satijalab.org/seurat/archive/v3.0/integration.html)^30^. Using 30 principal components, SPNs were clustered (*FindNeighbors*, *FindClusters*) using the original Louvain algorithm with a resolution of 0.8 and clusters were visualized using UMAP^4,31^. Using the clustering information for SPNs, subsets for dSPNs and iSPNs were further created. The dSPN and iSPN subsets were then subjected to pseudotime trajectory analysis using PHATE^20^. First, loom objects corresponding to dSPN and iSPN subsets were exported. Using loom objects as input and *scanpy*’*s* python implementation of PAGA/PHATE^20^ (https://scanpy-tutorials.readthedocs.io/en/latest/paga-paul15.html, https://scanpy.readthedocs.io/en/stable/generated/scanpy.external.tl.phate.html), pseudotime trajectories were computed using C17 cells as root populations. A UMAP visualization of cells colored by scaled diffusion pseudotime (DPT)^33^ was also generated. Pseudotime information for dSPNs and iSPNs was then used to align the trajectories using the ‘*cellAlign*’ approach^21^ (https://github.com/shenorrLab/cellAlign) and data were visualized using a heatmap accompanied with pseudotime densities. A similar approach was also used to perform sub-clustering and trajectory analysis of SPNs populations using cells from the P9 dataset as root population (Fig. S2B,C).

### Gene regulatory network analysis for SPNs

A list of mouse transcription factors (TFs) was obtained from a mouse tissue transcription factor atlas^34^. A unique list of 471 TFs falling into fetal brain and adult brain tissue categories were retained for gene regulatory network analysis using an *Arboreto* and *grnboost2* based approach^35^. First, raw counts corresponding to expressed (446/471) TFs was fetched separately for both dSPNs and iSPNs. A gene regulatory network (GRN) was built with raw expression data for dSPNs and iSPNs separately using python implementation of *Arboreto* and *grnboost2* (https://arboreto.readthedocs.io/en/latest/examples.html). The GRN output was then filtered following a previously published approach^35^, (https://github.com/bradleycolquitt/songbird_cells/tree/master/grn) to retain the top one percent of the TF-gene interactions, which were then visualized using the *igraph* R package (https://igraph.org/r/).

### Interneurons and oligodendrocytes sub-clustering and pseudotime trajectory analysis

Cell barcodes corresponding to clusters annotated as interneurons (*Pvalb*^+^,* Sst*^+^*/Npy*^+^,* Chat*^+^,* Calb2*^+^*/Th*^+^*)* were then used to subset from all the cells along with *Mki67*^+^ progenitors and *Sox4*^+^*/Sox11*^+^ neurogenic progenitors. Raw counts corresponding to the identified subset population for all datasets were then used to run Seurat integration approach (*FindIntegrationAnchors*, *IntegrateData*) to identify sub-populations among major interneuron categories (https://satijalab.org/seurat/archive/v3.0/integration.html)^30^. Using 30 principal components, interneuron cells were clustered (*FindNeighbors*, *FindClusters*) using the original Louvain algorithm with a resolution of 0.8 and clusters were visualized using UMAP^4,31^. The interneuron sub-clustering data were then subjected to pseudotime trajectory analysis using PHATE^20^. First, loom objects corresponding to interneuron clusters were exported. Using loom objects as input and scanpy’s python implementation of PAGA/PHATE^20^, (https://scanpy-tutorials.readthedocs.io/en/latest/paga-paul15.html, https://scanpy.readthedocs.io/en/stable/generated/scanpy.external.tl.phate.html), pseudotime trajectories were computed using cells expressing *Mki67* as root populations. UMAP visualization of cells colored by scaled diffusion pseudotime (DPT)^33^ was also generated. Gene expression patterns for specific sets of progenitor markers, interneuron markers and transcription factors were generated across scaled pseudotime. Similar to interneurons, sub-clustering and pseudotime trajectory analysis was also performed for the oligodendrocyte population including *Mki67*^+^ progenitors and a UMAP colored by scaled pseudotime was also generated.

### Mice

All experiments were approved by UT Southwestern IACUC # 2016-101-825. All methods were performed in accordance with all relevant guidelines and regulations as specified by UTSW IACUC and the American Veterinary Medical Association guidelines. This study is reported in accordance with ARRIVE guidelines. *Foxp1*^flox/flox^ mice were provided by Dr. Haley Tucker and backcrossed to C57BL/6J for at least 10 generations to obtain congenic animals as previously described^5^. *Drd1a-Cre* (262Gsat, 030989-UCD) and *Drd2-Cre* (ER44Gsat, 032108-UCD) mice were obtained from MMRC.

### Pseudobulk differential gene expression analysis for oligodendrocytes in P9 striatal scRNA-seq data

Oligodendrocyte clusters were identified based on known marker genes (*Olig1*+) and cells from each cluster were pooled by genotype (*Foxp1*^*D1*^, *Foxp1*^*D2*^, *Foxp2*^*DD*^, or *Foxp1*^*CTL*^). Differential expression was performed using the Poisson likelihood ratio test from Seurat R analysis pipeline between *Foxp1*^*CTL*^ and *Foxp1*^*D1*^, *Foxp1*^*D2*^, or *Foxp2*^*DD*^ oligodendrocytes. Significant expression cutoffs were adj. p-value ≤ 0.05 and abs(log_2_FC) > = 0.3.

### Protein isolation and immunoblotting

Striatal tissue from adult mice (P56) was harvested as previously described^5^. Briefly, tissue was flash frozen, and protein was extracted using 1X RIPA buffer (750 mM NaCl, 250 mM Tris–HCl pH7.4, 0.5% SDS, 5% Igepal, 2.5% Sodium deoxycholate, 5 mM EDTA, 5 mM NaVO4) with fresh protease inhibitor cocktail (10 μl/ml), 100 mM PMSF (10 μl/ml), and 200 mM sodium orthovanadate (25 μl/ml). Tissue was homogenized using a QIAGEN TissueLyser LT, rotated for 1 h at 4 °C, and spun down at max speed for 15 min. Protein was quantified using a standard Bradford assay and 20 μg of protein was loaded into a 10% SDS-Page gel. Protein samples were transferred to a PVDF membrane and then membrane was blocked in a 5% milk TBST solution. The following antibodies were used for immunoblots (IB) experiments: rabbit anti-MOBP (1:2000; Sigma HPA035152) or mouse anti-TUJ1 (1:10,000; Covance MMS-435P). Using an Odyssey infrared imaging system, rectangles were drawn around individual samples in either 800 or 700 IR channels to quantify the intensity signal after setting a background reference rectangle. MOBP signal was normalized to TUJ1 within each sample.

## Supplementary Information

Supplementary Figures.

## Data Availability

These data can be accessed and further analyzed through an interactive website (https://mouse-striatal-dev.cells.ucsc.edu).
