# Supplementary material for: A single-cell trajectory atlas of striatal development
Source: Sci Rep. 2023 Jun 3;13:9031. doi: 10.1038/s41598-023-36255-5 (PMC10239471; doi:10.1038/s41598-023-36255-5)
Supplement: Supplementary file 1 — Supplementary Figures. [file 41598_2023_36255_MOESM1_ESM.pdf]

Supplementary Information

Supplementary Figure Legends

Supplemental Figure 1. Comparison of genes across datasets and cell types. Comparison of detected genes across **(A)** individual datasets and **(B)** striatal cell types visualized by violin plots. **(C)** Stacked bar plot showing the contribution of cells from individual datasets across the 35 clusters and accompanied by violin plots showing the number of detected genes across striatal cell type clusters. **(D)** Scaled expression of marker genes for each cell type.

Supplemental Figure 2. cellAlign analysis of dSPNs and iSPNs. (A) Datasets plotted over pseudotime for SPNs, dSPNs (top), iSPNs (bottom) using C17 cells as root populations. **(B)** Datasets plotted over pseudotime for SPNs, dSPNs (top), iSPNs (bottom) using P9 cells as root populations. **(C)** Dissimilarity matrix and global alignment of pseudotime trajectories between iSPNs and dSPNs using cells from the P9 dataset as root.

Supplemental Figure 3. Uncropped Western blots of Figure 5E. Western blot results using antibodies for MOBP and TUJ1. Samples are imaged using an Odyssey infrared imaging system.

Figure S1

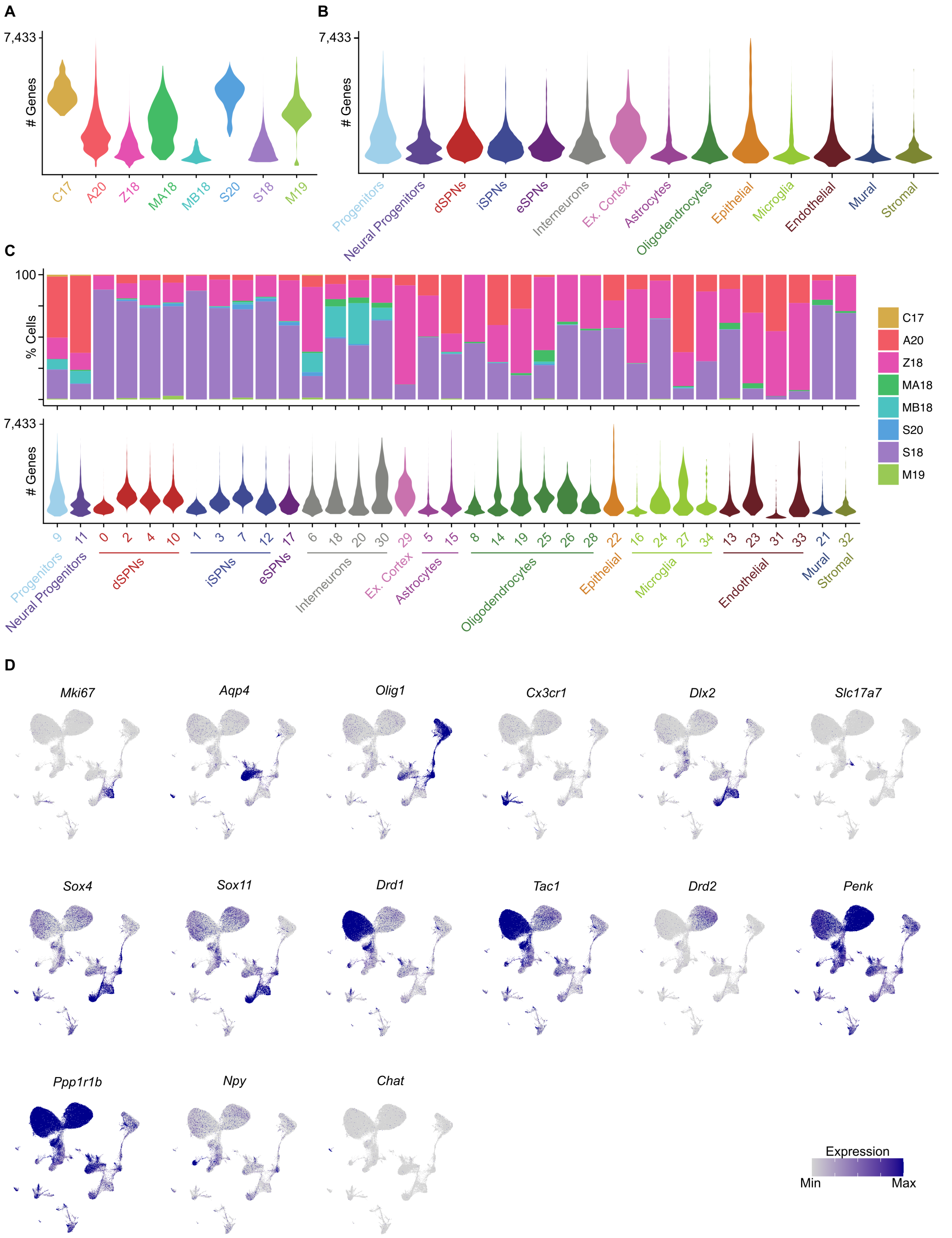
A
B
C
D
7,433
Genes
% Cells
100
C17
A20
Z18
MA18
MB18
S20
S18
M19
Progenitors
Neural Progenitors
dSPNs
iSPNs
eSPNs
Interneurons
Ex. Cortex
Astrocytes
Oligodendrocytes
Epithelial
Microglia
Endothelial
Mural
Stromal
9 11 0 2 4 10 1 3 7 12 17 6 18 20 30 29 5 15 8 14 19 25 26 28 22 16 24 27 34 13 23 31 33 21 32
Mki67
Aqp4
Olig1
Cx3cr1
Dlx2
Slc17a7
Sox4
Sox11
Drd1
Tac1
Drd2
Penk
Ppp1r1b
Npy
Chat
Expression
Min
Max

Figure S2

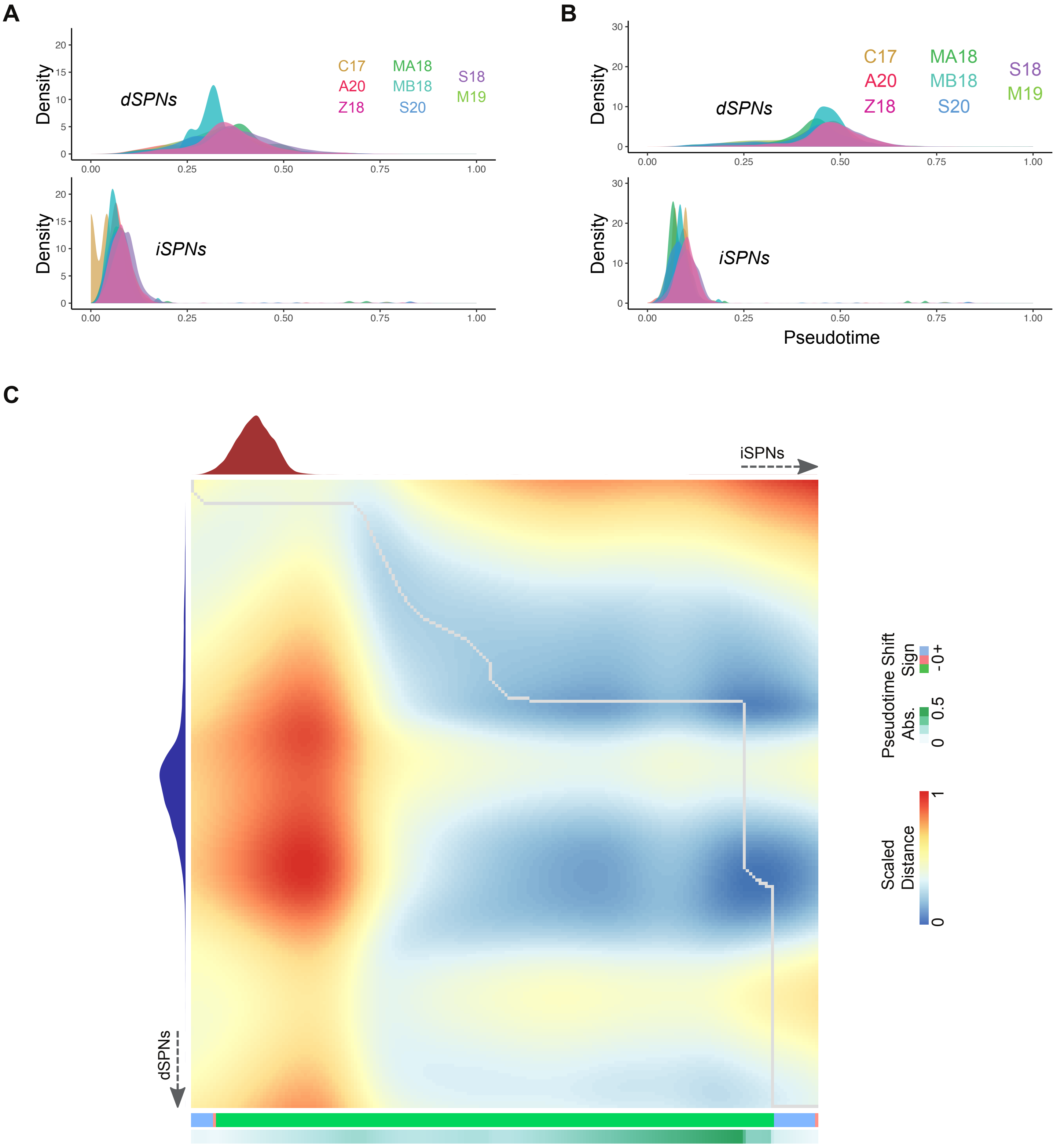

A
B
C
Density
dSPNs
iSPNs
C17
A20
Z18
MA18
MB18
S20
S18
M19
0.00
0.25
0.50
0.75
1.00
Pseudotime
iSPNs
dSPNs
Pseudotime Shift
Sign
-0+
Abs.
0
0.5
Scaled Distance
0
1

Figure S3

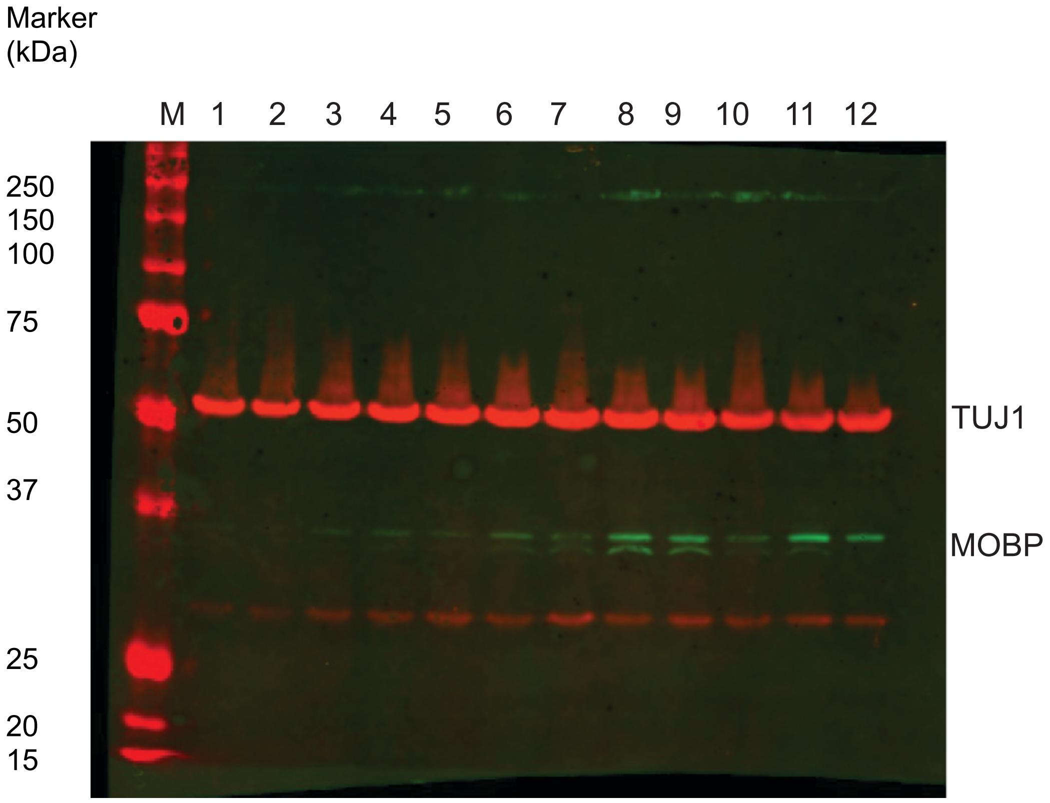

M= protein marker
1-3= *Foxp1*^{CTL} samples
4-6= *Foxp1*^{D1} samples
7-9= *Foxp1*^{D2} samples
10-12= *Foxp1*^{DD} samples
